# Breastfeeding, Breastmilk Feeding and Formula Feeding Within the Closed, Temporary Accommodation of Australia's COVID‐19 Quarantine

**DOI:** 10.1111/mcn.70271

**Published:** 2026-10-16

**Authors:** Michelle A. Fitzmaurice, Catharine Fleming, Kaitlyn Brunacci, Karleen D. Gribble

**Affiliations:** ^1^ St George & Sutherland Clinical Campuses, School of Clinical Medicine, Faculty of Medicine and Health University of New South Wales Sydney New South Wales Australia; ^2^ School of Medicine, Faculty of Health Western Sydney University Sydney New South Wales Australia; ^3^ School of Nursing and Midwifery, Faculty of Health Western Sydney University Sydney New South Wales Australia

**Keywords:** breastfeeding, child, COVID‐19, disasters, ill‐housed persons, infant, infant formula

## Abstract

During 2021–2022, families with infants and young children were among the > 450,000 people who spent 2 weeks in Australia's institutional COVID‐19 quarantine. Given pre‐existing lack of emergency planning for children, there were concerns about whether parents would be adequately supported in this temporary accommodation. This paper reports on research considering the breastfeeding, breastmilk feeding and formula feeding of infants and young children in Australia's COVID‐19 quarantine. Two‐hundred and sixteen parents who quarantined with children aged 0–36 months completed an online survey with closed‐ and open‐ended questions. One hundred and twenty‐seven children were being fed breastmilk (103) and/or infant formula (40) when entering quarantine. Breastfeeding, formula feeding and breastmilk feeding challenges were reported. Breastfeeding mothers were concerned about frequent feeding and stress reducing milk supply. Parents who were formula feeding commonly lacked appropriate resources with half having to wash feeding bottles in bathrooms that also contained a toilet. Mothers who were breastmilk feeding experienced the challenges associated with both breastfeeding and formula feeding. On‐site staff and health providers did not always respond to requests for help with appropriate assistance, and parents were rarely referred to health or parenting helplines. Parental capacity to follow the national infant feeding guidelines was commonly compromised by the quarantine environment, and child health was placed at risk. This study has implications for infant feeding resourcing and support in other types of temporary accommodation, including those related to natural disasters, family violence, or homelessness or any closed environment, such as prisons or immigration detention. Infant feeding support requirements should be included in emergency planning and standards developed for temporary accommodation for families.

## Key Points

- It should be assumed there will be infants in every population impacted by an emergency, and planning should be in place to ensure support for mothers and caregivers for safe infant and young child feeding practices.
- Access to breastfeeding counselling should be facilitated in any disaster or temporary accommodation situation.
- Formula‐fed infants and those dependent fully or partially upon expressed breastmilk should be considered vulnerable groups and any temporary accommodation their families are placed in must include kitchen facilities.
- Standards for temporary accommodation for families with infants and young children should be developed.

## 1 Introduction

The emergence of the SARS‐CoV‐2 virus resulted in numerous public health measures globally to minimise disease transmission. In Australia, institutional quarantine for interstate and international travellers formed a key component of the COVID‐19 pandemic response and in 2020 and 2021, more than 450,000 people completed 2 weeks in mandatory COVID‐19 quarantine (Halton 2021). Travellers were predominantly quarantined in hotels or serviced apartments but in the Northern Territory, a facility consisting of small cabins was used (Vivian 2022; Halton 2021). Among those required to quarantine were families with infants and young children, whose feeding and care needs had to be met within this temporary accommodation.

Australian national infant feeding policy is articulated in the National Health and Medical Research Council's Infant Feeding Guidelines (NHMRC Guidelines) (National Health and Medical Research Council 2013). The NHMRC Guidelines recommend infants be exclusively breastfed from birth until around 6 months of age, after which complementary foods should be introduced while breastfeeding continues until 12 months of age and beyond (National Health and Medical Research Council 2013). The NHMRC Guidelines state breastfeeding mothers should be provided with easily accessible breastfeeding support and counselling services. They additionally note that feeding of expressed breastmilk requires hot water and detergent for cleaning feeding equipment and refrigeration for when breastmilk is not fed immediately post‐expression (National Health and Medical Research Council 2013). The NHMRC Guidelines say that if an infant is not breastfed or is partially breastfed, they should be fed a commercial infant formula as a breastmilk substitute (National Health and Medical Research Council 2013). They outline that formula feeding requires safe water, a continuous supply of infant formula, ability to boil water for reconstitution, hot water and detergent for washing, and resources for sterilising (National Health and Medical Research Council 2013). The NHMRC Guidelines emphasise the importance of hygiene in the preparation of infant feeds, including that infant formula must be prepared in a clean area.

In Australia, direct breastfeeding, feeding expressed breastmilk, and formula feeding are all common infant feeding practices (Australian Bureau of Statistics 2022; Johns et al. 2016). The overwhelming majority of infants in Australia, > 90%, initiate breastfeeding (Australian Bureau of Statistics 2022). However, new mothers commonly experience breastfeeding challenges, including those related to low breastfeeding self‐efficacy and concerns about milk supply, infant fussiness, and frequent feeding, all of which may be exacerbated during emergencies (Summers and Bilukha 2018; Gribble et al. 2023). These challenges contribute to women introducing supplementary formula feeds or ceasing breastfeeding altogether in disasters (Summers and Bilukha 2018; Suzan et al. 2025; Gribble et al. 2023; Ratnayake Mudiyanselage et al. 2022). Provision of skilled breastfeeding counselling is therefore recognised as an essential component of emergency response to infants and young children in the Operational Guidance for Infant and Young Child Feeding in Emergencies (OG‐IFE) (IFE Core Group 2017).

Most breastfeeding mothers in Australia express breastmilk for their infants, including for reasons related to mother–infant separation, personal preference, or breastfeeding difficulties (Gridneva et al. 2025; Clapton‐Caputo et al. 2021). They do so at various intensities, from occasional to many times a day, and expressed breast milk may be fed exclusively, alongside direct breastfeeding, or in combination with infant formula (Gridneva et al. 2025; Clapton‐Caputo et al. 2021). Formula feeding is also common practice in Australia. Although national data are absent, existing evidence suggests almost all Australian infants are fed infant formula at some time, with most eventually being dependent on infant formula for all milk feeds (Magarey et al. 2015). In emergencies, access to physical resources for expressed breast milk and infant formula feeding may be compromised (Bartick et al. 2024). The OG‐IFE therefore states that targeted support should be provided to caregivers of formula‐fed infants (IFE Core Group 2017) while the Academy of Breastfeeding Medicine highlights the support requirements for expressed breast milk feeding in emergencies (Bartick et al. 2024).

Unfortunately, Australian emergency planning has overlooked the needs of infants and young children. An assessment undertaken by Save the Children in 2013 concluded that children suffer from “benign neglect” in emergency planning, with their “unique needs…not routinely and systematically considered” (Davie 2013, p. 7). A 2018 national audit of Australian emergency plans found “a dearth of planning at all levels of government for infants and young children,” including IYCF‐E (Gribble et al. 2019, p.7). These findings raised concerns that the Australian government may not have been supporting the feeding needs of infants and young children in COVID‐19 quarantine.

This study examined the breastfeeding, expressed breastmilk and formula feeding experiences of families with infants and young children during mandatory institutional COVID‐19 quarantine in Australia. The aim was to understand the challenges faced by parents in the milk feeding of their infants and young children within this temporary accommodation and the extent which they were supported or not to follow safe feeding practices as outlined in the NHMRC Guidelines.

## 2 Methods

Study participants were recruited from June to December 2021 via advertisements in Facebook (Meta Platforms Inc., Menlo Park, CA, USA) support groups for individuals experiencing COVID‐19 quarantine in Australia. Parents who had quarantined in an Australian COVID‐19 quarantine facility within the previous 6 months with a child aged 0–3 years were eligible to participate. Participants completed an online survey hosted on the Qualtrics platform. The survey included closed and open‐ended questions regarding parent and child demographics, quarantine facility characteristics, infant feeding and caregiving experiences, and wellbeing and safety. Quantitative data were analysed using descriptive statistics. Qualitative responses were analysed in NVivo using conventional content analysis (Hsieh and Shannon 2005). Ethical approval was provided by the University of Western Sydney Human Research Ethics Committee (approval H14452).

## 3 Results

Two hundred and sixteen parents; 205 (95%) mothers and 11 (5%) fathers ranging from 26 to 50 years of age (average 36 years) participated in the study. Study participants were highly educated with 174 (81%) having studied to at least degree level. Approaching half of study participants (92, 43%) quarantined in New South Wales (NSW), with Queensland the second most common jurisdiction of quarantine (40, 19%) (Table 1).

**Table 1:** Personal characteristics and jurisdiction of quarantine of study participants.

Characteristic	*N* = 216	%
Sex		
Female	205	95
Male	11	5
Age		
20–29	17	8
30–39	156	72
40–49	42	19
50+	1	0
Highest education achieved		
Year 10	1	0
Year 12	10	5
Certificate or trade	12	6
Diploma	15	7
Degree	79	37
Post‐graduate	95	44
Other	4	2
Quarantining jurisdiction		
New South Wales	92	43
Queensland Victoria	40	19
Western Australia Victoria	26	12
Victoria	24	11
Northern Territory	21	10
South Australia	11	5
Australian Capital Territory	2	1

Over one‐third of study participants (79, 37%) were in quarantine when they completed the survey. The vast majority (191/215, 89%) were quarantining after entering Australia from overseas, with the remainder (24/215; 11%) quarantining to travel between Australian jurisdictions. Almost half (101/215, 47%) were Australian citizens returning home after living overseas. The overwhelming majority (201, 93%) experienced general quarantine, whereas a small minority (13, 6%) experienced a “health” quarantine facility for individuals with medical conditions. One study participant quarantined twice with her children. Study participants were quarantined with between one and eight others (mean 2.7), and nearly one‐third (67/214) were mothers quarantining without another adult.

Two hundred and fifteen study participants provided information on the age of their children 0–36 months. Study participants were caring for 97 infants, of which 45 were 0–6 months, and 52 were 7–12 months. There were also participants caring for 75 children who were 13–24 months and 50 who were 25–36 months.

### 3.1 Breastfeeding, Breastmilk Feeding and Formula Feeding Patterns

One hundred and twenty‐seven children were receiving breastmilk (103 children) and/or infant formula (40 children). Twenty‐four children (19%) were receiving only breastmilk, six (5%) only infant formula, six (5%) breastmilk and infant formula, 63 (50%) breastmilk and complementary foods, 10 (8%) breastmilk, infant formula and complementary foods, and 18 (14%) infant formula and complementary foods.

Among the children receiving breastmilk, 69 (67%) were aged 0–12 months, 28 (27%) were 13–24 months, and six (6%) were 25–36 months. Almost all (97/103, 94%) received breastmilk through direct breastfeeding. Fourteen received breastmilk that has been expressed; eight children (8%) received both direct breastfeeding and expressed breastmilk, whereas six (6%) received only expressed breastmilk.

### 3.2 Breastfeeding Experiences

Breastfeeding mothers were asked to nominate the breastfeeding challenges they experienced during quarantine. As shown in Table 2, 77 (81%) experienced at least one challenge. The most reported difficulties were increased feeding frequency, concerns that stress had reduced their milk supply, painful nipples, low milk supply, and biting. One mother additionally reported inadequate food provision stating “Lack of food supplies for increased calories count. I was hungry and tired +++” [mother with a 2‐month‐old].

**Table 2:** Frequency of difficulties with breastfeeding in COVID‐19 quarantine.

Feeding difficulty	Frequency (n‐95)
Child waking more frequently to breastfeed overnight	47
Child wanting to breastfeed more frequently during the daytime	41
Stress reduced my milk supply	22
Painful nipples	12
Low milk supply	12
Biting	10
Blocked duct	8
Concerned about weight gain	7
Needing reassurance about breastfeeding	7
Mastitis	6
Breast refusal	5
Baby not attaching well	4
Too much milk	3
Child wanting to breastfeed less frequently	2
I did not experience any difficulties with breastfeeding	18

Fifteen breastfeeding mothers sought assistance for breastfeeding problems. Nine sought help from an onsite health provider, six from a friend or relative, six from the Australian Breastfeeding Association (ABA), and two from an offsite health provider. Experiences with onsite health staff varied considerably. One mother whose 3‐month‐old infant was refusing the breast and who was concerned about her milk supply reported the onsite doctor advised, “Don't use formula. She will learn her food comes from your boob, doesn't matter how long it takes?” [mother with a 3‐month‐old]. Another said she received, “Awful support from Metro Health nurses on call” [mother with a 4‐month‐old].

Some advice was inconsistent with infant feeding recommendations. One mother described how what the onsite nursing staff told her worked against her intention to follow recommended breastfeeding practices and undermined the support of her partner. She explained, “Although nurses very kind and helpful, they did not usually work with infants. I did not appreciate them telling me… that I should be starting solids already and that the guideline in Australia is for 4–5 months to start solids. This made my husband doubt my breastfeeding choices, despite me being 100% aware of the national and international recommendations for around 6 months exclusive BF” [mother with a 5‐month‐old]. In contrast, another study participant who experienced breastfeeding difficulties reported that she was able to access appropriate support via an onsite midwife.

When asked what their biggest challenge was caring for their child in quarantine, a number of participants described breastfeeding‐related issues. These included not having anywhere to sit to breastfeed apart from a bed (three study participants each with infants ≤ 2 months of age and quarantined in a single hotel room), concerns about a reduced milk supply (two study participants), and frequent feeding that was physically and emotionally demanding and undermined confidence (three study participants). Some participants described the psychological impact of quarantine as affecting their ability to care for their children, including breastfeeding. One woman quarantining alone with a breastfed 2‐month‐old infant and a 3‐year‐old toddler described being “sent to hospital for psychological assessment as I couldn't stop crying.” Another mother who was quarantining on her own with a breastfed 3‐month‐old and a 3‐year‐old described severe psychological distress saying she was “not coping,” “my brain is breaking.” She also expressed concerns that the quarantine conditions were affecting breastfeeding explaining, “Breast milk drying up due to stress of inappropriate conditions…Dry nappy overnight when usually is very full. Orange urine.” In response to this concerning information, one of the researchers contacted the hotel to advise that women with infants should have access to appropriate health support, including referral to the ABA National Breastfeeding Helpline. The Offices of the relevant state Ministers for Health and Police were also contacted and advised of concern that mothers of infants were not being adequately supported in quarantine.

Seven study participants described breastfeeding as being the thing that helped them most in feeding and caring for their children. One mother said, “Breastfeeding made it easy to have instant access to food” [mother of a 7‐month‐old]. Conversely, other mothers reported breastfeeding frequently to be physically and mentally difficult. One study participant described, “I wish I had not had to breastfeed the entire time…my son wanted to feed from me all day and all night!” [mother of an 18‐month‐old].

### 3.3 Formula Feeding

The 40 formula‐fed infants of study participants were in 37 families (three sets of twins), and at least one formula feeding challenge was reported for half (20, 50%) of these infants (Table 3). Many formula feeding challenges were regarding difficulties safely preparing formula feeds. These included because there were inadequate or absent resources for washing and sterilising or no suitable place to prepare formula feeds or to store infant formula and feeding equipment.

**Table 3:** Frequency of difficulties with formula feeding in COVID‐19 quarantine.

Feeding difficulty	Frequency *n* = 40
Facilities for washing were not good enough	16
Facilities for sterilising were not good enough	14
Place for preparing formula feeds was not good enough	12
Lack of a suitable place to store formula and feeding equipment	11
Difficulty getting infant formula	8
Baby wanting formula feeds more frequently	3
Baby refusing formula feeds	3
Concerned about weight gain	2
Baby wanting formula feeds more frequently during the daytime	1
Needed reassurance about feeding	1
I did not experience any difficulty formula feeding my baby	20

Infant formula was prepared in a range of locations with just over half of study participants doing so in a kitchen (21/40, 53%), 15 (38%) in a living room or bedroom, and seven (18%) in a bathroom containing a toilet. Study participants also used a variety of locations to wash feeding equipment; 19 (51%) washed feeding bottles in a bathroom sink proximate to a toilet, 15 (41%) used a kitchen sink, two (5%) used a dishwasher, and one (3%) used a bucket. Thirty percent (11/37) of families with a formula‐fed infant were quarantined in a single hotel room, and for 20 parents (54%), the only place they had to wash feeding equipment was in a bathroom containing a toilet.

Just over half of formula‐feeding parents (20/36, 56%) reported sterilising feeding bottles while in quarantine. Sterilisation methods included boiling bottles inside a kettle (6, 17%), using a microwave steam steriliser (5,14%), chemical sterilisation (3, 8%), and boiling bottles in water on a stovetop (1, 3%). Three parents (8%) described their sterilisation method as involving placing bottles in a bowl and adding boiling water, while one mother described soaking bottles in hot water in the garbage bin in her room. Eight out of 15 parents (53%) who did not sterilise feeding bottles indicated this was because they lacked equipment to do so.

The jurisdiction of quarantine influenced the extent to which parents faced challenges with formula feeding, as this influenced whether they had access to a kitchen or not. Just over half (111/216, 51%) of study participants had access to a kitchen in quarantine. However, this varied substantially by jurisdiction: 96% of study participants quarantining in NSW had access to a kitchen with a kitchen sink compared to 0%–25% in the other jurisdictions (excluding the ACT in which just two participants quarantined) (Table 4). Thus, formula feeding parents outside of NSW reported feeding challenges at almost double the rate of those who were quarantined in NSW (18/26, 69% vs. 5/14, 36%).

**Table 4:** Quarantine facilities with a kitchen sink by jurisdiction.

Jurisdiction	Number of study participants by jurisdiction	Quarantine facilities with a kitchen sink *n* (%)
NSW	92	88 (96)
Qld	40	10 (25)
WA	26	4 (15)
Vic	24	5 (21)
NT	21	0 (0)
SA	11	2 (18)
ACT	2	2 (100)
Total	216	111 (51)

Thirteen study participants sought assistance for formula feeding challenges. Of these, seven sought assistance from an on‐site health provider, five from a friend or relative and one from an off‐site health provider. Assistance requests most often included washing and sterilising equipment, but appropriate resources were not always provided. For example, “Requested a microwave to sterilise bottles and was denied. Was told I would get a steriliser, but it didn't happen. They brought me a bucket and told me to boil the water for the bottles. The next day they brought me sterilising tablets to use in the bucket. The bucket had a hole in it and leaked 4 Litres of water on the table and floor” [mother with a 2‐month‐old].

Obtaining infant formula was a challenge faced by parents of 20% (8/40) of formula‐fed infants. One mother described, “I had to wait a long time for online delivery. The hotel did not provide any food for a 9‐month‐old!!!” [mother with a 9‐month‐old]. Another said, “My husband (not in quarantine) had to ring the department to authorise the delivery of milk formula for my son through special permission… It was the hardest and stressful 24 h as no one would help us” [mother with a 9‐month‐old].

Some participants relied on help from family or friends when appropriate support or resources were unavailable. One participant described requesting a bowl to wash feeding bottles, explaining, “Asked for a salad bowl or similar to wash bottles in…was told we had to wash them in the toilet sink and no other options could be provided. Had family deliver a bowl after hotel and health team refused to provide ‐ this took several days to organise and had to use dirty bottles in the meantime” [mother with a 9‐month‐old].

When asked an open question about their biggest challenge in feeding and caring for their children in quarantine, difficulties associated with formula feeding were described by some study participants. This included in relation to problems obtaining infant formula. For example, “Having to make 8 calls in 1 day to get infant formula delivered” [mother with a 9‐month‐old]. Being required to wash feeding equipment in bathrooms with a toilet was described by several parents as their biggest challenge: “Hygienic conditions washing bottles in bathroom with no real sterilising option [mother with a 2‐month‐old],” “Washing bottles next to the loo was vile” [mother with an 11‐month‐old] and “Being sanitary with bottle preparation and washing when the sink is in bathroom, 1 m from toilet. This is not an acceptable quarantine facility for families” [mother with a 2‐month‐old]. Figure 1 is a photograph of the facilities available to one mother for washing her breast pump parts and feeding bottles.

**Figure 1:**
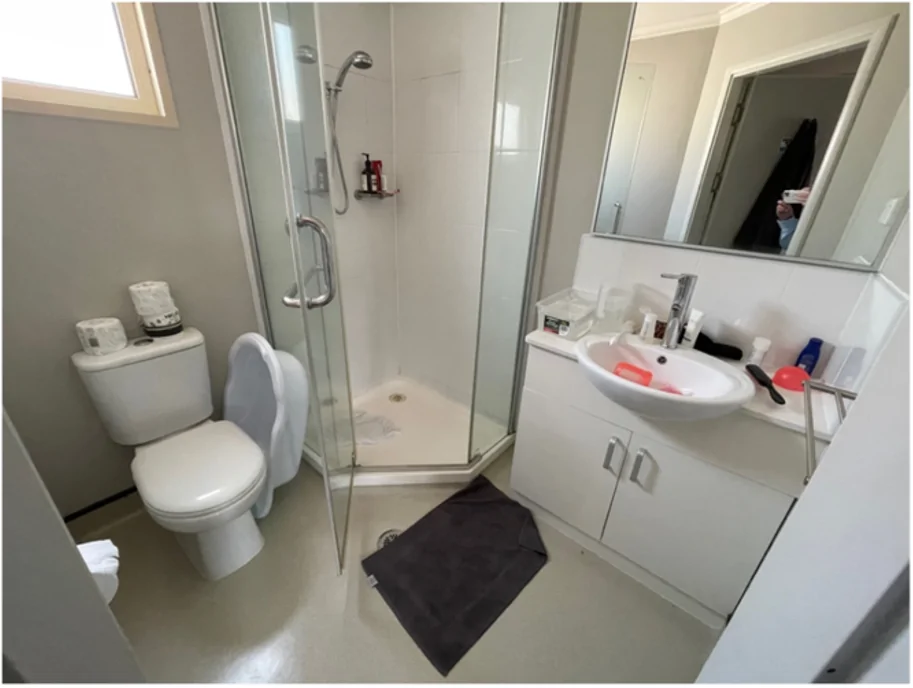
The washing facilities available to a mother in the Howard Springs Centre who was expressed breastmilk and formula feeding a 3‐month‐old infant.

### 3.4 Breastmilk Feeding Experience

Twelve study participants who were feeding their infants expressed breastmilk (86%) reported experiencing at least one feeding difficulty (Table 3). The most commonly reported challenges were not having adequate facilities for washing breast pump parts and bottles, concerns that stress had reduced milk supply, infants wanting to feed more frequently, and low milk supply.

Participants reported washing breast pump parts in a variety of locations; six participants (46%) used a kitchen sink, four (31%) a sink in a bathroom with a toilet, three (23%) a bucket or other container, and one (8%) a sink in a bathroom without a toilet. Inadequate refrigeration for storing expressed breastmilk was also reported; one participant explained there was “No facility to safely store expressed milk, milk was going off, bar fridge set to 7 degrees” [mother of a 9‐month‐old].

Four participants sought assistance for expressed breastmilk feeding challenges; two sought assistance from a friend or relative, two from an on‐site health provider, one from an off‐site health provider and one from the ABA. One participant described relying on her mother to provide resources for cleaning breast pump parts and feeding equipment because the supplies provided by the quarantine venue were inadequate. She explained, “Spoke to Metro Health team about… lack of facilities to clean bottles… I called hotel reception to request a tub for washing up and a larger kettle to heat the water…but the tub and kettle they supplied were too small to be of any use… my mum [dropped off] a plastic tub I could use for washing up, a large kettle for heating water, bottle brush and some tea towels” [mother with a 3‐month‐old].

### 3.5 Referral to Infant Feeding Support Services

One hundred and twelve participants provided information on referrals to health and parenting support services. Only six (5%) reported being referred to any external health or parenting service; four (4%) to the ABA National Breastfeeding Helpline, three (3%) to the Health Direct Helpline and one (1%) to the Pregnancy, Birth and Baby Helpline.

## 4 Discussion

This research identified challenges to the safe breastfeeding, formula feeding and breastmilk feeding of infants and young children were frequent in the temporary accommodation of Australia's COVID‐19 quarantine. Breastfeeding challenges reported by women in quarantine were not novel or unusual and should have been expected and planned for. They included painful nipples, mastitis, breast refusal and the need for reassurance; all common reasons why Australian mothers seek breastfeeding support (Kam and Haines 2021). Many study participants had concerns about frequent feeding and stress reducing their milk supply which as previously stated, is not unusual in disasters. Although there is no known mechanism by which physiological stress can directly reduce milk production, stress can reduce oxytocin release and slow milk ejection (Ueda et al. 1994). This may result in infants being unsettled at the breast, feeding for shorter or longer periods, or breastfeeding more frequently. Children may also breastfeed more frequently during difficult times because breastfeeding provides comfort (Gribble 2008a; Gribble 2010; Waters et al. 2014). Jetlag, disrupted sleep patterns and close mother‐child proximity may all have contributed to more frequent breastfeeding and maternal concerns about milk supply in quarantine (Gribble 2008b; Markovic et al. 2021).

While the NHMRC Guidelines and the OG‐IFE (IFE Core Group 2017) emphasise the importance of providing breastfeeding counselling to mothers, appropriate breastfeeding support seemed not easily available within the quarantine system. Furthermore, mothers were rarely referred to telephone support services that could have assisted them. In particular, lack of referral to the Australian Government‐funded 24‐h ABA National Breastfeeding Helpline was a significant oversight.

Few environmental barriers to breastfeeding were identified. The exception was three mothers with young infants who were quarantined in a single hotel room and who didn't have somewhere comfortable to breastfeed. Given individual breastfeeds in young infants may last up to an hour and occur eight to fourteen times a day this lack should not be considered insignificant (Kent et al. 2012).

In contrast to breastfeeding, challenges faced by formula feeding parents were primarily environmental. These included inadequate facilities for storing feeding supplies, preparing formula feeds and washing and sterilising feeding bottles. Quarantine authorities which placed families in facilities without a kitchen appeared not to have considered what resources parents needed to safely feed their children and to lack recognition of the risks poor hygiene posed to these infants. Infant formula may be intrinsically contaminated (Fusi et al. 2025) or may be contaminated through handling of the infant formula scoop (Cho et al. 2019) or during feed preparation (Marino et al. 2007). Infant formula is a good medium for bacterial proliferation and bacterial biofilms may form on the surfaces and crevices of infant feeding equipment (Iversen et al. 2004). Hence, the requirements of health guidance globally (e.g. WHO & FAO 2007; United States Centers for Disease Control and Prevention 2024; National Health Service 2023) including within the NHMRC Guidelines (National Health and Medical Research Council 2013), for formula feeds to be prepared in a clean location and for feeding equipment to be thoroughly washed and sterilised.

Preparing formula feeds in bathroom containing a toilet. or washing feeding bottles in a bathroom sink also used for washing hands after toileting presents clear hygiene concerns. Nevertheless, parents of approximately half the formula fed infants in this study reporting washing feeding equipment in these sinks, often without access to sterilising equipment. Consequently, the quarantine accommodation did not provide some parents with facilities to follow the Australian government guidance for the safe preparation of infant formula (National Health and Medical Research Council 2013).

Mothers expressing breastmilk experienced both the breastfeeding‐related challenges associated with stress, concerns about milk supply and frequent feeding, and the environmental barriers encountered by formula‐feeding parents. While breast pump kits may be more difficult to clean than bottles due to their complex design, antibacterial properties of breast milk provide some protection against bacterial growth (Rodrigo et al. 2021; Sosa and Barness 1987). Nonetheless, inadequate facilities for cleaning breast pump equipment and safe storage of expressed breast milk are also unacceptable.

Only quarantining authorities in NSW appear to have recognised the additional needs of families with young children and seem to have preferentially accommodated them in apartments rather than hotel rooms. This decision was a systemic substantive action protective of the ability of parents to safeguard their child's health and follow the NHMRC Guidelines. Unfortunately, there is no publicly available information explaining why or how this decision was come to. In contrast, the decision of the other jurisdictions to routinely place families with infants in facilities without a kitchen reflects a systemic failure. Neither of the Australian government reviews of COVID‐19 quarantine identified any of the issues raised in this research or any concerns regarding infants generally (Halton 2021; Australian Government 2020). This perhaps reflects a more general invisibility of infants and young children in Australian disaster inquiries that needs to be addressed (Gribble 2026b).

These findings have broader relevance. While the pandemic context was unusual, many families experience temporary accommodation during natural disasters, or due to family violence, or homelessness (Batterham et al. 2023; Nowicki et al. 2019; Nowicki 2023; Watts et al. 2018; Pehi et al. 2025; Kuskoff et al. 2025; Hartley and Barnes 2023). While research regarding infants in temporary accommodation is extremely limited, a lack of kitchen and washing facilities have been identified as problems for families in temporary accommodation in Australia and the United Kingdom (Batterham et al. 2023; Kuskoff et al. 2025; Feldman 2013). Prisons and immigration detention are additional “closed” settings where mothers may also be required to care for their infants and young children, and support there may also be lacking. For example, it was recently identified that incarcerated mothers in Australia have very limited access to the ABA National Breastfeeding Helpline (Gribble 2026a).

### 4.1 Limitations

Study participants were a convenience sample recruited through online Facebook support groups and were very highly educated. Study participants may not have been atypical for COVID‐19 quarantine, as it would be expected that the expatriates and international travellers required to quarantine would be of above average education and socioeconomic status. However, it would be expected that in other temporary accommodation situations, families would be much less advantaged on average. They therefore would be more likely to be formula feeding and have less financial and social resources to mitigate against challenges and may experience difficulties not identified in this study. Further research is needed to understand the infant feeding challenges and support needs of families in other forms of temporary accommodation.

## 5 Conclusions

This study comprises the first research to consider infant feeding experiences in a temporary accommodation setting in any depth and highlights system‐level gaps and barriers relevant to future emergency and temporary accommodation planning. The breastfeeding challenges reported by mothers were largely consistent with those found in other emergency settings and reflected a need for breastfeeding counselling. Formula feeding and expressed breastmilk feeding were largely affected by environmental barriers especially inadequate facilities for cleaning and sterilisation as well as supply chain issues. Families were not uncommonly placed in a position where they were unable to follow government guidelines for formula feeding or feeding expressed breastmilk. These findings have implications beyond the COVID‐19 pandemic and highlight the importance of incorporating infant feeding support requirements into emergency preparedness and temporary accommodation planning. It should not be considered acceptable children to be placed in temporary accommodation that poses a risk to them and standards for temporary accommodation for families should be developed.

## Author Contributions

MF, CF and KG conceived of and designed the study. MF, KB and KG analysed the data. All authors contributed to the writing and critical review of the paper.

## Conflicts of Interest

The authors declare no conflicts of interest.

## Data Availability

The data that support the findings of this study are available on request from the corresponding author. The data are not publicly available due to privacy or ethical restrictions.
